# Machine learning in the prediction of post-stroke cognitive impairment: a systematic review and meta-analysis

**DOI:** 10.3389/fneur.2023.1211733

**Published:** 2023-08-03

**Authors:** XiaoSheng Li, Zongning Chen, Hexian Jiao, BinYang Wang, Hui Yin, LuJia Chen, Hongling Shi, Yong Yin, Dongdong Qin

**Affiliations:** ^1^Department of Rehabilitation Medicine, The Affiliated Hospital of Yunnan University, Kunming, China; ^2^Department of Research and Teaching, Lijiang People’s Hospital, Lijiang, China; ^3^Department of Rehabilitation Medicine, The Third People’s Hospital of Yunnan Province, Kunming, China; ^4^Key Laboratory of Traditional Chinese Medicine for Prevention and Treatment of Neuropsychiatric Diseases, Yunnan University of Chinese Medicine, Kunming, China

**Keywords:** cognitive impairment, prediction, machine learning, stroke, meta-analysis

## Abstract

**Objective:**

Cognitive impairment is a detrimental complication of stroke that compromises the quality of life of the patients and poses a huge burden on society. Due to the lack of effective early prediction tools in clinical practice, many researchers have introduced machine learning (ML) into the prediction of post-stroke cognitive impairment (PSCI). However, the mathematical models for ML are diverse, and their accuracy remains highly contentious. Therefore, this study aimed to examine the efficiency of ML in the prediction of PSCI.

**Methods:**

Relevant articles were retrieved from Cochrane, Embase, PubMed, and Web of Science from the inception of each database to 5 December 2022. Study quality was evaluated by PROBAST, and c-index, sensitivity, specificity, and overall accuracy of the prediction models were meta-analyzed.

**Results:**

A total of 21 articles involving 7,822 stroke patients (2,876 with PSCI) were included. The main modeling variables comprised age, gender, education level, stroke history, stroke severity, lesion volume, lesion site, stroke subtype, white matter hyperintensity (WMH), and vascular risk factors. The prediction models used were prediction nomograms constructed based on logistic regression. The pooled c-index, sensitivity, and specificity were 0.82 (95% CI 0.77–0.87), 0.77 (95% CI 0.72–0.80), and 0.80 (95% CI 0.71–0.86) in the training set, and 0.82 (95% CI 0.77–0.87), 0.82 (95% CI 0.70–0.90), and 0.80 (95% CI 0.68–0.82) in the validation set, respectively.

**Conclusion:**

ML is a potential tool for predicting PSCI and may be used to develop simple clinical scoring scales for subsequent clinical use.

**Systematic Review Registration:**

https://www.crd.york.ac.uk/prospero/display_record.php?RecordID=383476.

## 1. Introduction

Stroke is a serious condition and a leading cause of death and long-term disability, which places a huge burden worldwide (1). Post-stroke cognitive impairment (PSCI) is a prevalent prognosis and cause of death following a stroke. Stroke patients have a higher incidence of 1-year cognitive impairment than non-stroke populations (2, 3). As society and economy progress, more emphasis is placed on disease and health, especially cognitive impairment. Early identification and diagnosis of PSCI, as well as early prophylaxis and treatment, can help improve stroke patient’s prognosis and reduce social and economic burdens.

Clinical tools for early PSCI diagnosis in stroke patients are currently lacking. Researchers have tried to apply existing cognitive impairment risk prediction models constructed based on the general population to the prediction of PSCI, but their predictive performance was not ideal in stroke patients (4). As a result, researchers have shifted their focus to machine learning (ML) in the hopes of developing more accurate PSCI prediction models. ML is an emerging field in medicine that utilizes computer science and statistics to solve healthcare problems (5). In recent years, ML has been increasingly applied to stroke research, and it was shown that ML-based stroke image prediction can outperform existing prediction tools (6). However, the diversity in mathematical modeling and sensitivity of ML algorithms to factors such as patient sampling, missing data and sample size continue to fuel debates over the accuracy of these models in disease prediction.

The performance of existing stroke prediction models has been inconsistent due to the use of different types of ML (e.g., logistic regression or other alternative) and modeling variables. In these predictive models, there are differences in the types of machine learning utilized, with most researchers using logistic regression while some may consider it lacking and opt for alternative models. Furthermore, we note discrepancies in the selection of modeling variables, which ultimately contributes to the uncertainty of their results. Unfortunately, evidence-based studies investigating the efficiency of ML in the prediction of PSCI are still lacking. As a result, the aim of this study is to examine the predictive accuracy of ML in PSCI and comprehensively summarize the modeling variables included in this prediction model in order to provide a useful reference for the subsequent development of simple clinical prediction tools.

## 2. Materials and methods

This study was conducted following the Preferred Reporting Items for Systematic Reviews and Meta-Analyses guidelines (Supplementary material) (7).

This study has been registered in PROSPERO (CRD42022383476).

### 2.1. Eligibility criteria

#### 2.1.1. Inclusion criteria

1. Patients diagnosed with ischemic stroke or hemorrhagic stroke.
2. Randomized-controlled trials (RCTs), case–control studies, cohort studies, and case-cohort studies.
3. Complete construction of a ML prediction model for PSCI.
4. Studies without external validation are also included.
5. Different studies published using the same data set.
6. Studies reported in English.

#### 2.1.2. Exclusion criteria

1. Meta-analysis, review, guidelines, and expert opinions.
2. Only risk factor analysis was performed and lacks a complete ML model.
3. Missing outcome measures (ROC, c-statistic, c-index, sensitivity, specificity, accuracy, recovery rate, accuracy rate, confusion matrix, diagnosis table, F1 score, and calibration curve).
4. Validation of the maturity scale only.
5. Study on the accuracy of single-factor prediction models.

### 2.2. Search strategy

Relevant articles were systematically searched in Cochrane, Embase, PubMed, and Web of Science from the inception of each database to 5 December 2022 using MeSH and entry terms without restriction on language or region. The detailed retrieval process is outlined in Supplementary material.

### 2.3. Literature screening and data extraction

Retrieved articles were imported into Endnote for management, and duplications were deleted. The titles and abstracts were screened to exclude irrelevant studies, and the full texts of the remaining records were downloaded and checked for eligibility. Data were collected from the included studies using a customized data extraction form. The collected data comprised title, first author, year of publication, author country, type of study, source of patient, type of stroke, diagnostic criteria for cognitive impairment, length of follow-up, number of PSCI cases, total subject number, training set, validation set, type of model used, imputation method for missing value, variable screening, and modeling variables. Two independent researchers (YY and HY) performed the literature screening and data extraction, and subsequently cross-checked their results. Any disagreement was resolved by a third researcher (XSL).

### 2.4. Risk of bias assessment

The Prediction model Risk Of Bias Assessment Tool (PROBAST) was employed to evaluate the quality of the included studies. The PROBAST consists of four domains, namely participants, predictors, outcome, and analysis (8). The four domains contain 2, 3, 6, and 9 specific questions, respectively. Each question has three options: yes/probably yes (Y/PY), no/probably no (N/PN), and no information (NI). If a domain has at least one N/PN, it is rated as high risk. To be graded as low risk, a given domain must have Y/PY for all questions. When all domains are at low risk, the overall risk of bias is low; alternatively, when at least one domain is assessed as high risk, the overall risk of bias is high (9). Two researchers (XSL and DDY) independently evaluated the risk of bias in the included studies and subsequently cross-checked their results. Any disagreement was resolved by a third researcher (BYW).

### 2.5. Outcome measures

The primary outcome was the C-index, which can be used to reflect the overall accuracy of ML models. However, this indicator alone may not fully reflect the predictive accuracy of ML models in PSCI because the percentage of PSCI patients and non-PSCI patients in the included literature is severely unbalanced. Therefore, sensitivity and specificity were included as complementary outcome measures to evaluate the predictive accuracy of ML in PSCI.

### 2.6. Data synthesis and statistical analysis

The c-index and accuracy of ML models were meta-analyzed. If a 95% confidence interval (CI) and standard error were missing for the c-index, they were estimated using the methods by Debray (10). Given the differences in modeling variables and parameters, the c-index was pooled using a random effects model while sensitivity and specificity were pooled by a bivariate mixed effects model. In systematic reviews based on machine learning, heterogeneity is difficult to avoid. According to the Cochrane tool, percentages of around 25% (I^2^ = 25), 50% (I^2^ = 50), and 75% (I^2^ = 75) are deemed to represent low, medium, and high levels of heterogeneity, respectively (11). The sensitivity and robustness of the results were evaluated using the leave-one-out method. Publication bias was qualitatively assessed using a funnel plot and quantitatively assessed by Egger’s regression test (value of *p*). All meta-analyses were conducted in R4.2.0 (R development Core Team, Vienna, http://www.R-project.org). A *p* < 0.05 was considered statistically significant.

## 3. Results

### 3.1. Study selection

**Figure 1:**
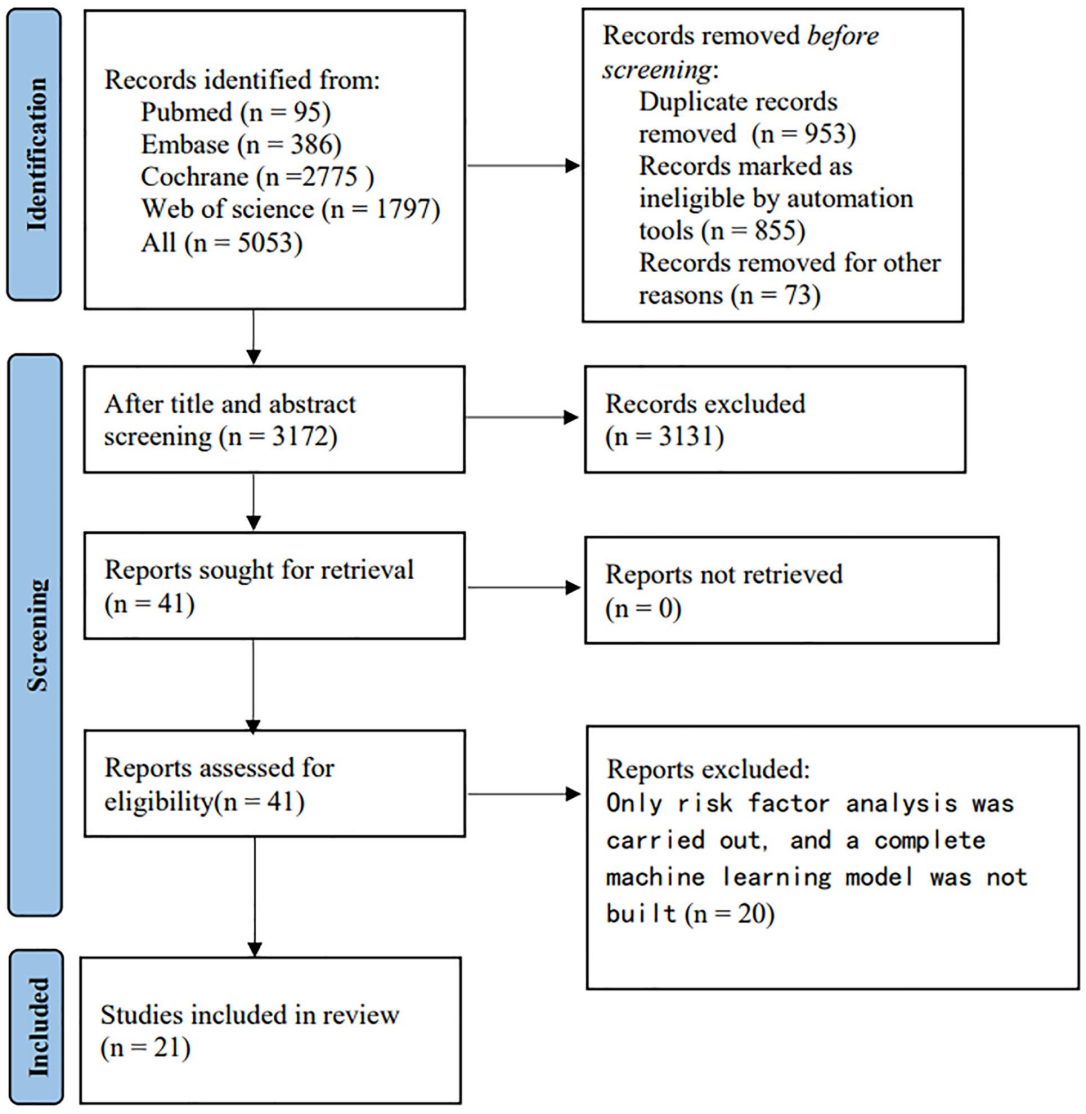
Flowchart of study selection.

The literature screening process is illustrated in Figure 1. We identified a total of 5, 053 unique records. After reviewing the full texts of 41 reports, 21 studies were ultimately included (12–32).

### 3.2. Characteristics of included studies

Of the 21 eligible studies, 10 were conducted in China (12, 13, 17, 19, 21, 25, 26, 28, 29, 32), 2 in Norway (14, 27), 1 in Australia (20), 2 in German (15, 16), 2 in the Republic of Korea (18, 30), 1 in the Netherlands (14), 1 in France (23), 1 in the UK (24), and 1 in Singapore (31). These studies were published between 2015 and 2023, predominantly in 2021–2023 (*n* = 17) (12–27, 32).

Of the 7,822 subjects in the included studies, 2,876 developed PSCI. The subject cohort size ranged from 72 to 22,950. The diagnostic criteria used in the included studies were Mini-Mental State Examination (MMSE) (33), Montreal Cognitive Assessment (MoCA) (34) (*n* = 5) (20, 21, 28, 29, 31), MMSE (*n* = 6) (12–14, 18, 24, 32), MoCA (*n* = 6) (17, 19, 22, 25–27), Center of Cancelation (CoC) (35) (*n* = 1) (15), Global Deterioration Scale (GDS) (36) (*n* = 1) (16), Informant Questionnaire on Cognitive Decline in the Elderly (IQCODE) (37) (*n* = 1) (23), and vascular dementia criteria of the AHA/ASA scientific statement (38) (*n* = 1) (30). The duration of follow-up was predominantly 3 to 12 months, and was 36 years in only one study (23). The incidence of PSCI during follow-up was 12.5%–66.1%.

### 3.3. Characteristics of included prediction models

There were 31 models in the included studies, which were constructed based on logistic regression (LR) nomogram (*n* = 18) (12, 14–16, 18, 19, 22, 25, 26, 29–32), random forest (*n* = 1) (20), ridge regression (*n* = 1) (23), LASSO regression (*n* = 1) (21), mixed effects model (*n* = 3) (24), support vector machine (SVM) classifier (*n* = 3) (27), and decision trees (*n* = 3) (28) (Table 1). Modeling variables were selected using a multivariate approach. In the training set, 15 models reported c-index and 13 models reported sensitivity and specificity. In the validation set, 10 models reported c-index and 7 models reported sensitivity and specificity. The main modeling variables used in the included studies were age, gender, education level, stroke history, stroke severity, lesion volume, lesion site, stroke subtype, and vascular risk factors (Table 2).

**Table 1:** General characteristics of included studies.

No.	First author	Year of publication	Author country	Study type	Souce of patients	Stroke type	Diagnostic criteria for cognitive impairment
1	Yinwei Zhu (12)	2022	China	RCT	Multicenter	Acute ischemic stroke	MMSE < 25
2	Fei Zha (13)	2022	China	Retrospective cohort study	Single center	Cerebral stroke	MMSE score ≤ 19 (illiteracy), ≤ 22 (primary education), ≤ 26 (Secondary school and above)
3	Georgios Vlachos (14)	2023	Norway	Retrospective cohort study	Multicenter	Mild acute stroke	the Barthel ADL index and the modified Rankin Scale (mRS).
4	Lisa R¨ohrig (15)	2022	Germany	Prospective cohort study	Multicenter	Right hemisphere stroke	letter cancelation test; bells cancelation test the Center of Cancelation [CoC; (35)]; The CoC
5	Ragnhild (16)	2022	Norway	Prospective cohort study	Multicenter	Cerebral stroke	Premorbid cognitive status based on GDS
6	Zhao-Yin Ma (17)	2022	China	RCT	Single center	Acute ischemic stroke	MoCA score ≥ 26 indicates normal cognitive function; < 26 indicates MCI; < 20 indicates CI
7	Reeree Lee (18)	2021	Republic of Korea	Prospective cohort study	Multicenter	Cerebral stroke	Objective neuropsychology tests, including MMSE and CDR
8	Yongzhe Gu (19)	2022	China	Retrospective cohort study	Multicenter	Ischemic stroke	MoCA score < 26
9	Nacim Betroun (20)	2022	Australia	Prospective cohort study	Multicenter	Cerebral stroke	MMSE score < 27 or MoCA score < 25
10	Xueling Yuan (21)	2021	China	Retrospective cohort study	Single center	Cerebral stroke	MMSE score ≤ 17 (illiteracy), ≤ 20 (Primary education), ≤ 24 (Secondary school and above), MOCA score ≤ 26
11	Nick A Weaver (22)	2021	Netherlands	Retrospective cohort study	Multicenter	Cerebral stroke	Performance below the fifth percentile of local normative data in at least one cognitive domain on the Multidomain Neuropsychological Assessment or the Montreal Cognitive Assessment
12	Renaud Lopes (23)	2021	franc	Retrospective cohort study	Multicenter	Cerebral stroke	IQCODE 49 ± 2
13	Youssef Hbid (24)	2021	UK	Prospective cohort study	Single center	First occurrence of cerebral stroke	MMSE score < 24 or AMT < 8
14	Li Gong (25)	2021	China	Prospective cohort study	Multicenter	Mild acute stroke	MoCA score < 22
15	Yi Dong (26)	2021	China	Retrospective cohort study	Multicenter	Acute ischemic stroke	MoCA score < 22
16	Eva Birgitte Aamodt (27)	2021	Norway	Prospective cohort study	Multicenter	Cerebral stroke	TMT A and B, CERAD, COWAT, MoCA, AD-8, GDS (36), NPI Q, HADS, and the Cornell scale
17	Yueli Zhu (28)	2020	China	Prospective cohort study	Multicenter	First occurrence of cerebral stroke	MMSE score < 27 and MoCA score < 21
18	Zhengbao Zhu (29)	2019	China	Prospective cohort study	Multicenter	Ischemic stroke with elevated blood pressure	MMSE score < 27 or MoCA score < 25
19	Jae-Sung Lim (30)	2017	Republic of Korea	Prospective cohort study	Multicenter	Cerebral stroke	AHA-ASA Criteria, at least two cognitive defects
20	Nagaendran Kandiah (31)	2015	Singapore	Retrospective cohort study	Multicenter	Mild acute ischemic stroke	MMSE score ≤ 2 5 or MoCA score ≤ 22
21	Sheng Ye (32)	2022	China	Retrospective cohort study	Single center	Lacunar infarction	MMSE score < 24

Follow-up duration	Number of patients with cognitive impairment after stroke	Total sample size	Number of patients with cognitive impairment in training set	Total sample size of training set (deriving set and modeling cohort)	Validation set generation method [internal validation (k-fold cross-validation, leave-one-out method, random sampling), external validation (prospective, institutional)]	Number of patients with cognitive impairment in validation set	Total sample size of validation set	Variable screening/feature selection method	Model type
3 m	228	599	228	599	No validation set			Multivariate	Logistic regression
3 m	87	367	58	245	Random sampling at a ratio of 2:1	29	122	Multivariate	Logistic regression
12 m	21	117	21	117	No validation set			Multivariate	Logistic regression
	27	103	27	103	No validation set			Multivariate	Logistic regression
3 m	91	589	91	589	No validation set	No	No	Multivariate	Logistic regression
	94	161	94	161	No validation set	No	No	Multivariate	Logistic regression
6 m	19	110	19	110	Random internal validation	12	70	Multivariate	Logistic regression
6 m	69	123	69	123	External validation	38	60	Multivariate	Logistic regression
1y	77	327	62	262	5-fold cross validation	15	65	Multivariate	random forest
1y	118	376	118	376	External validation	75	125	Multivariate	LASSO regression
			106	338	10-fold cross validation	12	38		
15 m	1,286	2,950	1,286	2,950	External validation	107	246	Multivariate	Logistic regression
			1,179	1,704					
					12-fold cross validation				
36y	9	72	9	72	External validation		40	Multivariate	Ridge regression
5y	1,000	2,468	1,000	2,468	External validation	204	940	Multivariate	Mixed effects model
					Internal validation				
1y	112	228	112	228	External validation	No	1,000	Multivariate	Multivariate logistic regression
6 m	131	383	131	383	External validation		281	Multivariate	Multivariate logistic regression
					Internal validation		102		
3 m	125	227	125	227	Leave-one-out cross validation		227	Multivariate	Classifier model
6 m	66	104	66	104	Internal cross validation	66	104	Multivariate	Decision tree
3 m	340 MMSE scoring	638			No			Multivariate	Logistic regression
	422 MoCA scoring								
3 m	50	308			No			Multivariate	Logistic regression
6 m	78	209	70	88	10-fold cross validation	8	21	Multivariate	Logistic regression
			78	209	External validation	35	185		
12 m	52	313	38	219	Random sampling at a ratio of 7:3	14	94	Multivariate	Logistic regression

MMSE, mini-mental state examination; CDR, clinical dementia rating; GDS, global deterioration scale; MoCA, Montreal cognitive assessment; IQCODE, informant questionnaire on cognitive decline in the elderly; AMT, abbreviated mental test; TMT A and B, neuropsychological test battery included Trail making A and B; CERAD, 10 word memory and recall test; COWAT, the controlled oral word association test; AD-8, ascertain dementia 8-item informant questionnaire; NPI Q, neuropsychiatric inventory; HADS, hospital anxiety and depression scale.

**Table 2:** Modeling variables in the included studies.

Variables	Frequency
Age	29
Gender	26
Educational level	24
Diabetes	15
Smoking	15
Hypertension	14
History of stroke	11
Stroke severity	11
Lesion size	10
Hyperlipemia	9
Drinking	9
Stroke subtype	8
Body mass index	8
NIHSS	7
Site of lesion	7
Race	7
Coronary heart disease	6
NIHSS score	6
Atrial fibrillation	6
MoCA (baseline)	5
MMSE (baseline)	5
Antiplatelet before stroke	5
Glycosylated hemoglobin (HbA1c)	5
Information about cognitive decline in the elderly	4
homocysteine	4
Number of days after stroke	4
APOE ε4 positive	3
Fazekas	3
mRS (baseline)	3
sTREM2	3
WMH size	3
Fluvastatin	3
Hypercholesteremia	3
Family history of stroke	3
Transient ischemic attack, a prestroke vascular risk factor	3
Uroclepsia	3
Cortical thickness	3
Cognitive examination	3
Socioeconomic group	3
Use of antihypertensive drugs	3
Cardiovascular risk factors	3
aCL	2
aPS	2
DS-WMH	2
HS	2
PV-WMH	2
RF	2
β2-GPi	2
Imaging time (days after the event)	2
The time from disease onset to randomization	2
Red blood cell distribution width	2
Mean corpuscular volume	2
Diastolic blood pressure	2
Random treatment	2
Hemoglobin	2
Scan sequence or pattern for infarct segmentation	2
Hypoglycemic drugs	1
Comorbidity2	1
Fazekas score	1
FBG	1
FDG PET DL-based cognitive assessment	1
hsCRP	1
IQCODE score	1
LDL-C	1
MTA pathology	1
NINDS-CSN 5-minute protocol score	1
NLR	1
OCSP classification	1
SAA	1
Imaging features of T1-weighted (T1w) image texture analysis	1
OCSP classification and functional level in advanced TOAST	1
TOAST classification	1
White blood cell count	1
White matter hyperintensity	1
Premorbid cognitive decline	1
At discharge (NIHSS, mRS, Barthel scores)	1
Low density lipoprotein	1
Triglyceride	1
Cysteine proteinase inhibitor	1
Country	1
Marital status	1
Progression of acute stroke	1
Memory	1
Employment situation	1
Anticoagulant drugs	1
Infections treated with antibiotics	1
1 point for six or more correct answers	1
Hexagonal orientation	1
Number of intracranial atherosclerotic stenosis	1
Incranial volume	1
Chronic lacunes	1
Uric acid	1
Global cortical atrophy and stenosis of large intracranial vessels	1
Presence of any APOE-e4 allele	1
Visual space function	1
Affected vascular area	1
Pentaterial memory	1
Pre-existing depression	1
Fibrinogen	1
Myocardial infarction	1
Serum albumin	1
Language	1
Speech fluency raw score	1
Executive function	1
Stroke classification	1
Stroke feature	1
Neutrophil-lymphocyte ratio (NLR)	1
NCD	1

### 3.4. Risk of bias assessment

**Figure 2:**
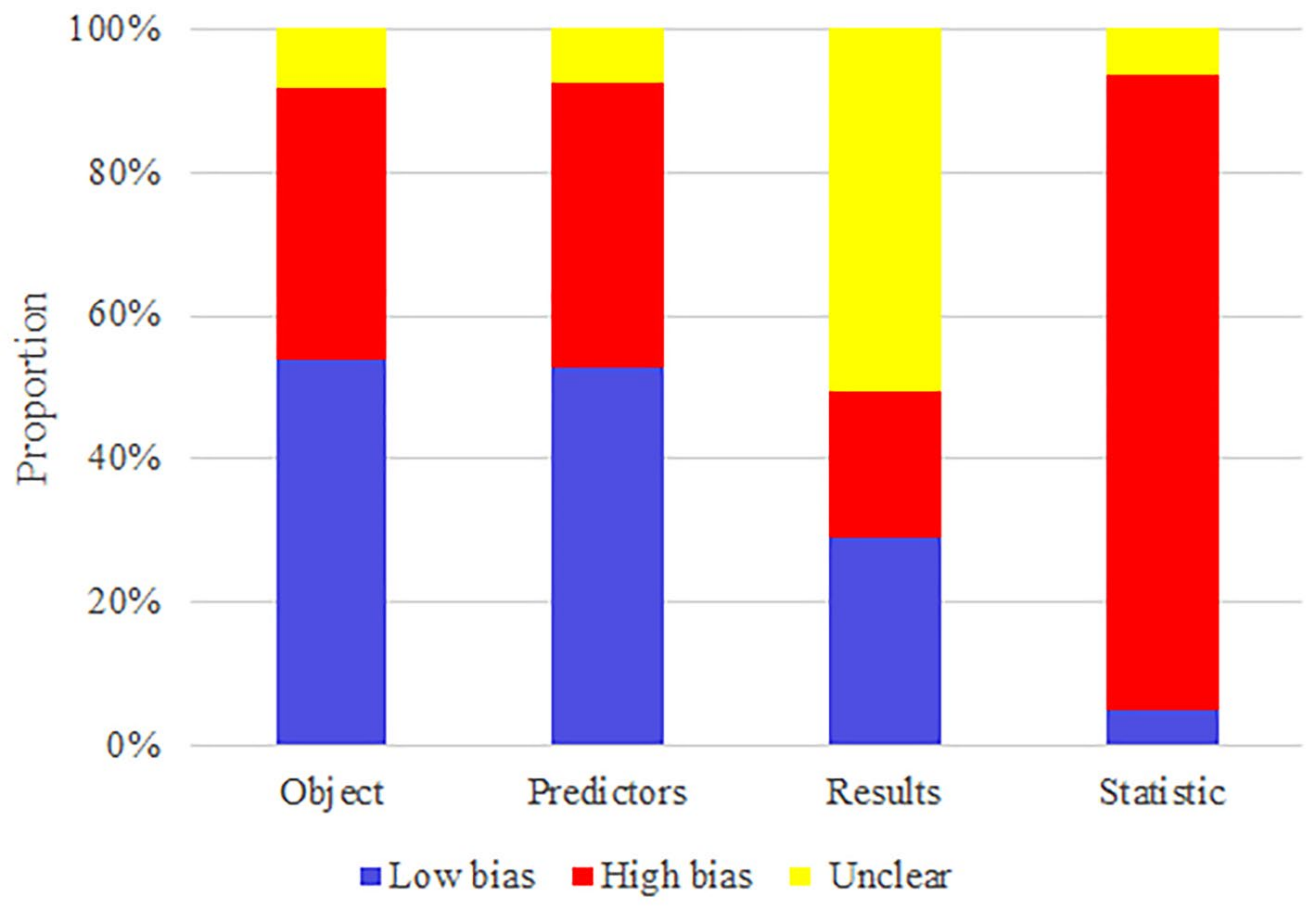
Risk of bias assessment.

The high risk of bias in the included studies was attributed to the limited sample size, retrospective cohort study, and lack of validation set. Therefore, these attributes should be improved in subsequent model construction. The results of the risk of bias assessment are summarized in Figure 2.

### 3.5. Meta-analysis

**Figure 3:**
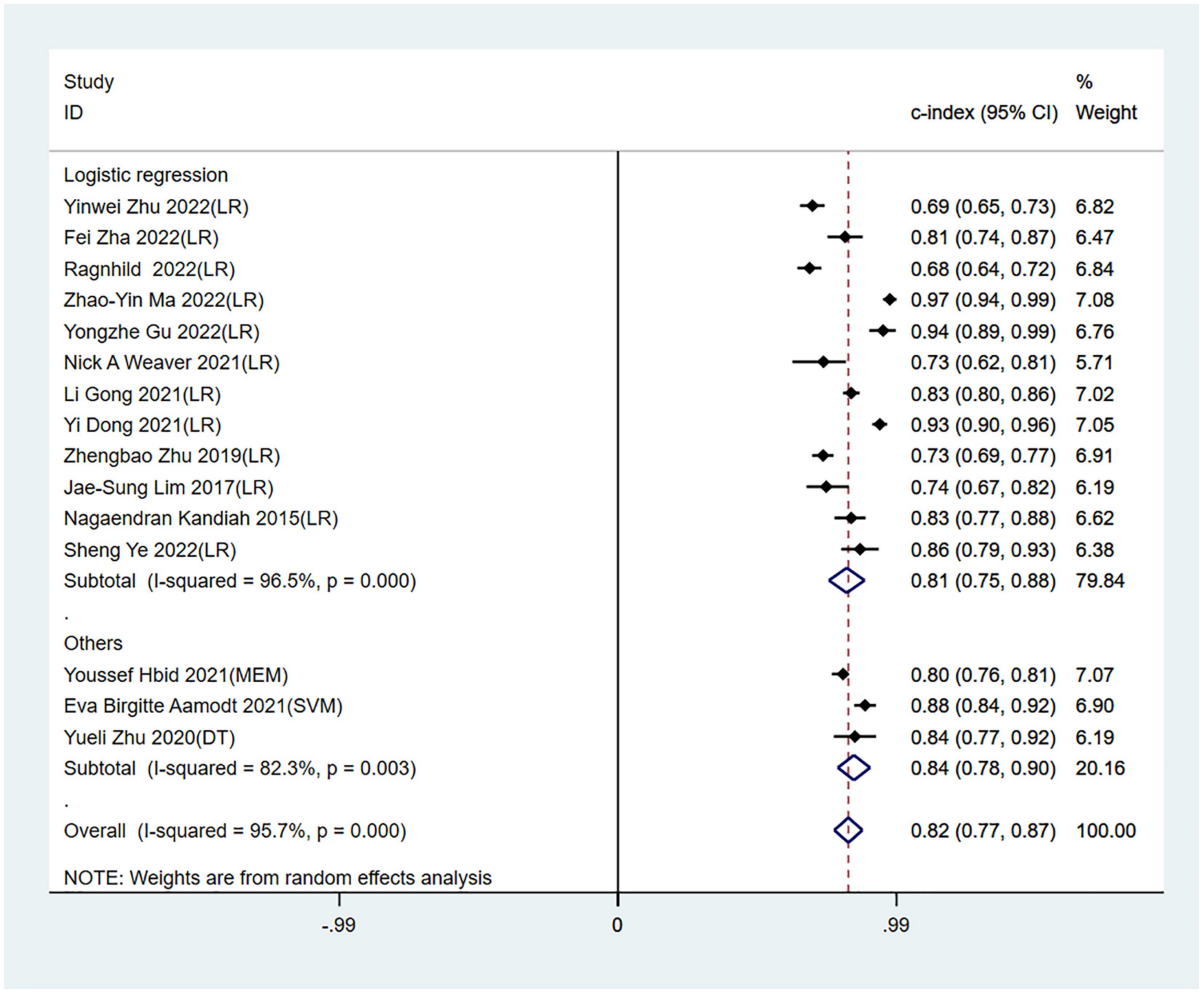
Forest plot of c-index in the training set. LR, logistic regression; SVM, Support Vector Machines; DT, decision trees; MEM, mixed effects model.

**Figure 4:**
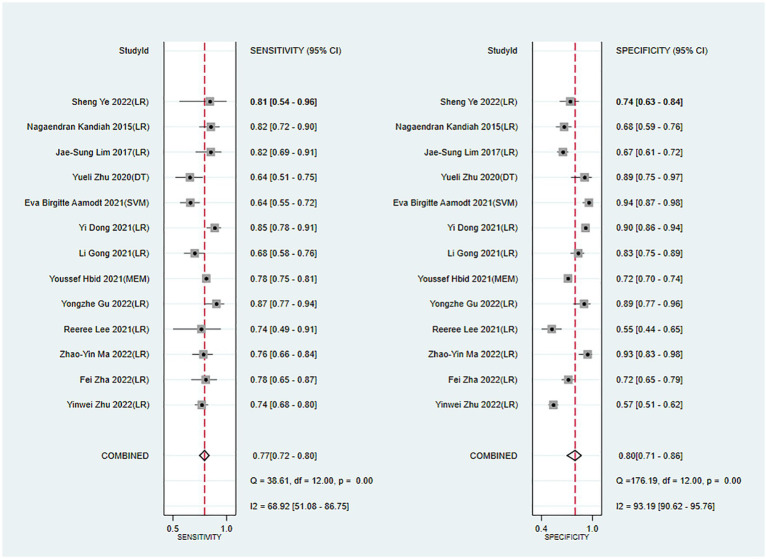
Forest plot of sensitivity and specificity in the training set. LR, logistic regression; SVM, Support Vector Machines; DT, decision trees; MEM, mixed effects model.

Meta-analysis showed that the training set had a c-index of 0.82 (95% CI 0.77–0.87, *n* = 15), sensitivity of 0.77 (95% CI 0.72–0.80, *n* = 13), and specificity of 0.80 (95% CI 0.71–0.86, *n* = 13). Subgroup analysis of the training set showed that the c-index was 0.81 for LR (95%CI 0.74–0.88, *n* = 12), 0.80 for mixed effects model (95%CI 0.76–0.81 *n* = 1), 0.88 for SVM classifier (95%CI 0.84–0.92 n = 1), and 0.84 for decision trees (95%CI 0.77–0.92 *n* = 1; Figures 3, 4).

**Figure 5:**
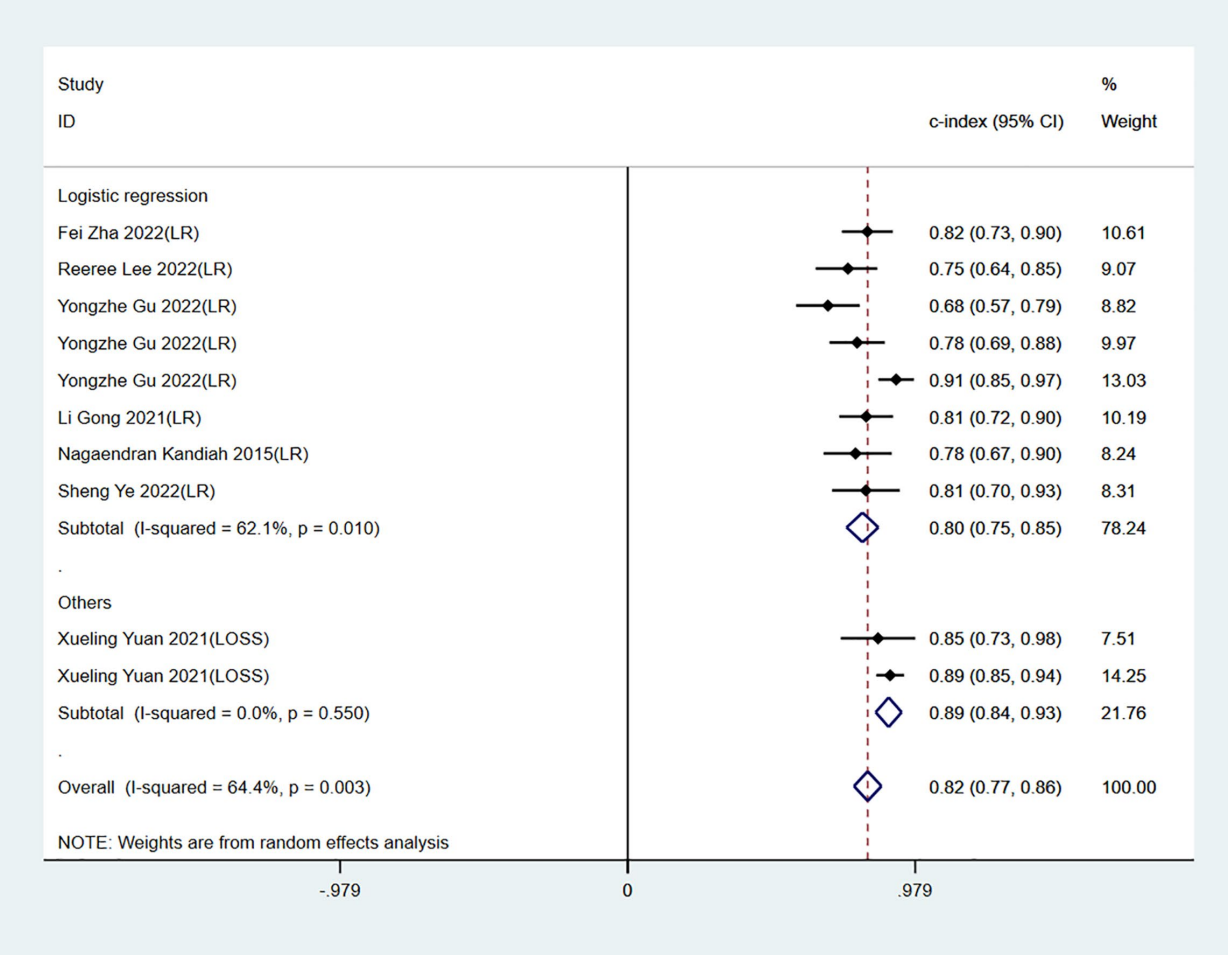
Forest plot of c-index in the validation set. LR, logistic regression; LASSO, least absolute shrinkage and selection operator.

**Figure 6:**
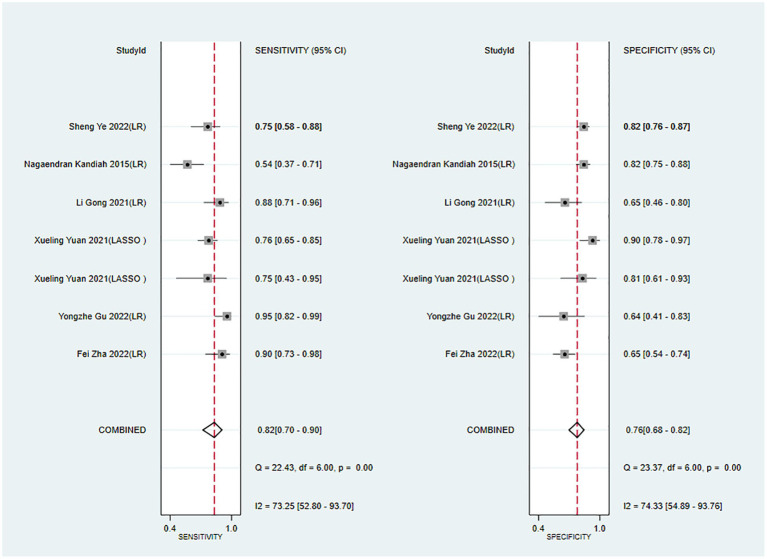
Forest plot of sensitivity and specificity in the validation set. LR, logistic regression; LASSO, least absolute shrinkage and selection operator.

The c-index, sensitivity, and specificity of the validation set were 0.82 (95% CI 0.77–0.87, *n* = 10), 0.82 (95% CI 0.70–0.90, *n* = 7), and 0.76 (95% CI 0.68–0.82, *n* = 7), respectively. Subgroup analysis of the validation set showed that the c-index was 0.80 for LR (95%CI 0.75–0.85, *n* = 8) and 0.89 for LASSO regression (95%CI 0.84–0.93 *n* = 2; Figures 5, 6).

**Figure 7:**
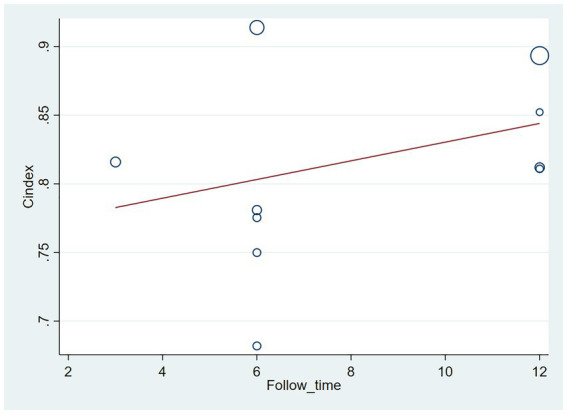
Meta-regression bubble plot of follow-up time in the training set (circles represent weights, with larger circle indicating greater weight and smaller confidence interval).

**Figure 8:**
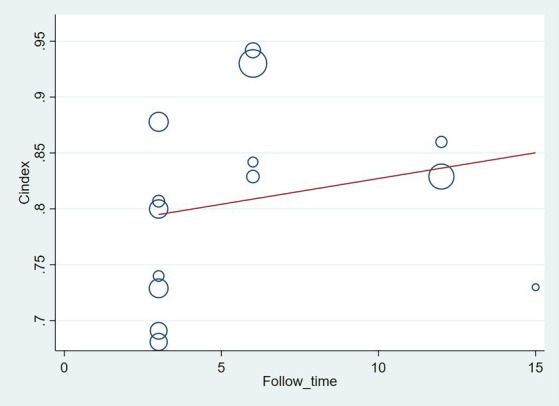
Meta-regression bubble plot of follow-up time in the validation set (circles represent weights, with larger circle indicating greater weight and smaller confidence interval). (1) Randomized controlled trial; (2) Prospective cohort study. (3) Retrospective cohort study.

**Figure 9:**
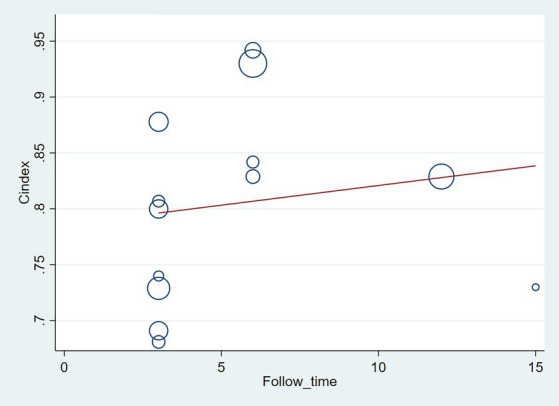
Meta-regression bubble plot of design in the training set (circles represent weights, with larger circle indicating greater weight and smaller confidence interval. (1) Randomized controlled trial; (2) Prospective cohort study; (3) Retrospective cohort study).

**Figure 10:**
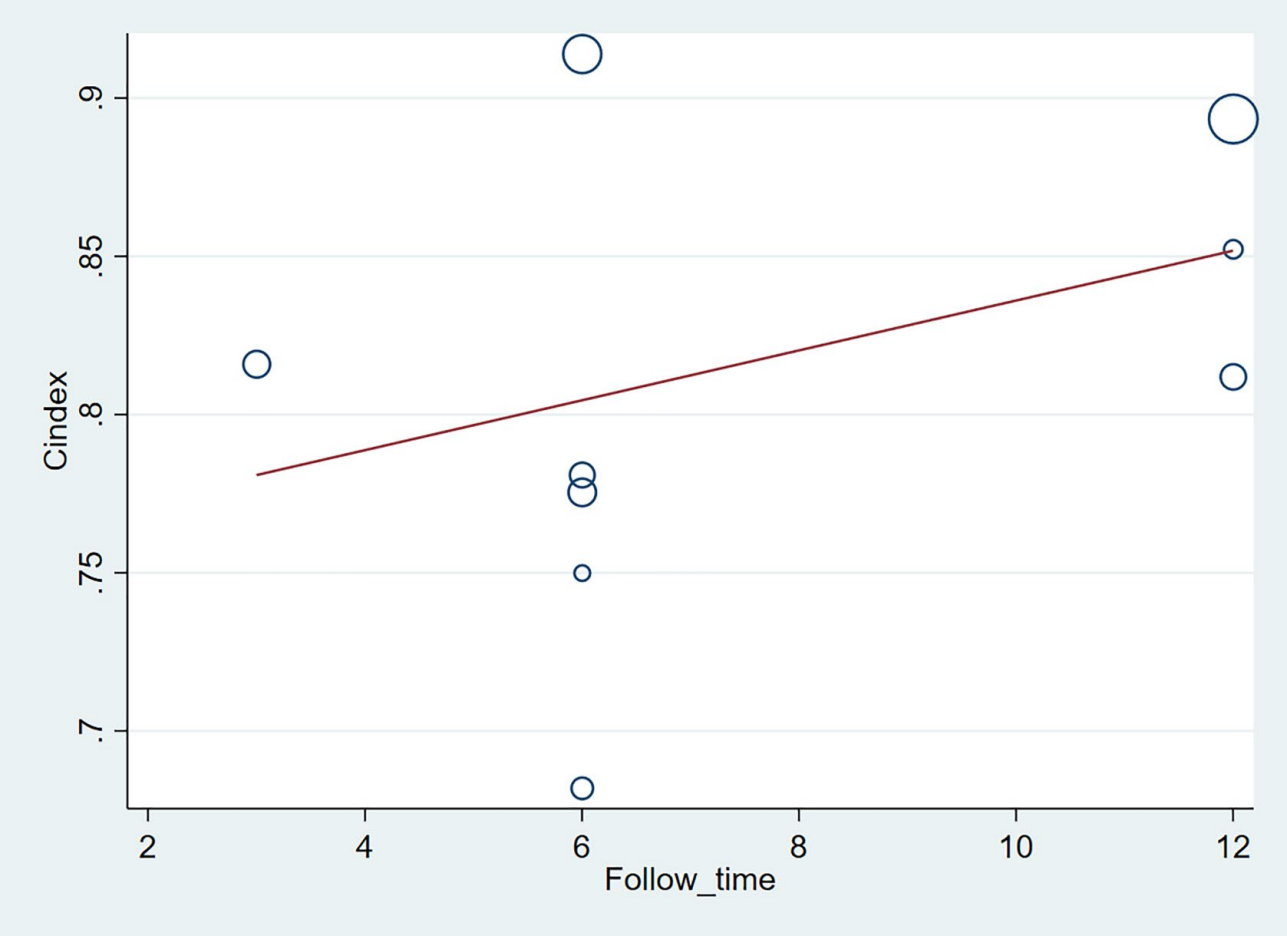
Meta-regression bubble plot of design in the validation set (circles represent weights, with larger circle indicating greater weight and smaller confidence interval).

The follow-up period or the meta-regression based on study design showed that there were no significant differences in the c-index between the training and validation sets, even considering the variations due to different study designs or changes in follow-up time (Figures 7–10; Tables 3, 4).

**Table 3:** Meta-regression results (follow-up time) in the training and validation sets.

Meta-regression					REML estimate of between-study variance		% residual variation due to heterogeneity		Proportion of between-study variance explained			
Number of obs					tau2		I-squared_res		Adj R-squared			
Training sets			Validation sets		Training sets	Validation sets	Training sets	Validation sets	Training sets	Validation sets		
14			10		0.00649	0.003267	93.53%	63.72%	−1.07%	−2.04%		
**With Knapp-Hartung modification**												
**Cindex**	**Coefficient**		**Std. err**		**t**		**P > t**		**[95% conf. interval]**			
	**Training sets**	**Validation sets**	**Training sets**	**Validation sets**	**Training sets**	**Validation sets**	**Training sets**	**Validation sets**	**Training sets**	**Validation sets**	**Training sets**	**Validation sets**
Follow_time	0.0046061	0.0068074	0.0060551	0.0070785	0.76	0.96	0.462	0.364	−0.0085867	−0.0095157	0.0177989	0.0231306
_cons	0.7810826	0.7623026	0.0420074	0.0619474	18.59	12.31	0.000	0.000	0.6895563	0.6194516	0.8726088	0.9051536

**Table 4:** Meta-regression results (study design) in the training and validation sets.

Meta-regression					REML estimate of between-study variance		% residual variation due to heterogeneity		Proportion of between-study variance explained			
Number of obs					tau2		I-squared_res		Adj R-squared			
Training sets			Validation sets		Training sets	Validation sets	Training sets	Validation sets	Training sets	Validation sets		
15			10		0.007951	0.003363	95.86%	63.50%	−3.08%	−5.03%		
**With Knapp-Hartung modification**												
**Cindex**	**Coefficient**		**Std. err**		**t**		**P > t**		**[95% conf. interval]**			
	**Training sets**	**Validation sets**	**Training sets**	**Validation sets**	**Training sets**	**Validation sets**	**Training sets**	**Validation sets**	**Training sets**	**Validation sets**	**Training sets**	**Validation sets**
Design	0.0265053	0.0195741	0.0353083	0.0297913	0.75	0.66	0.466	0.530	−0.0497738	−0.0491247	0.1027843	0.088273
_cons	0.7595276	0.7680337	0.0828655	0.0785483	9.17	9.78	0.000	0.000	0.5805077	0.586901	0.9385476	0.9491663

### 3.6. Sensitivity analysis and publication bias

Sensitivity analysis indicated that the results of both the training and validation sets were robust (Supplementary Figures S1, S2). However, the asymmetry in the funnel plot and the results of Egger’s regression test suggest that publication bias may be present in the training set (*p* = 0.056 for Egger’s regression test), and publication bias is clearly present in the validation set (*p* = 0.005 for Egger’s regression test; Supplementary Figures S3–S6). There were fewer independent validation cohorts in the included literature, and the presence of multiple independent validation cohorts in the same study may have contributed to publication bias.

## 4. Discussion

Our meta-analysis of 21 original studies demonstrated that ML may be an ideal tool for predicting PSCI. The training set had a c-index of 0.82 (95% CI 0.77–0.87) and sensitivity and specificity of >70%, indicating considerable predictive accuracy in PSCI. Furthermore, the accuracy of the validation set was not significantly lower than that of the training set, indicating that the ML model has good applicability. Currently, LR is the preferred model in clinical practice because it is simple for generating highly accessible nomograms, such as the nomogram on lymph node metastasis developed by the Sloan-Kettering Cancer Center (39–41). In our study, LR was also the preferred model among researchers as it exhibited comparable c-index performance to other ml algorithms while achieving higher sensitivity and specificity. As a result, we conclude that LR demonstrates satisfactory predictive ability for PSCI in this study.

We found that LR is the primary type of model utilized for predicting stroke. LR is a classification algorithm that aims to establish the relationship between features and probability of specific outcomes (42). ML is commonly used to address issues encountered in clinical practice, with supervised learning and unsupervised learning being the most common approaches. Supervised learning primarily focuses on diagnosing and predicting disease prognosis or progression, which involves the process of training, validation, and testing. The training process involves inserting predictive factors into the model and using the model’s inherent parameter calculation rules (e.g., maximum likelihood estimation, iteration) to estimate the optimal model parameters. Selection of modeling variables (feature selection methods) is crucial for the training process and has been a subject of ongoing debate due to their diversification. Furthermore, validation and testing are crucial for a completed model as they reflect the model’s robustness. Unfortunately, in actual research, most studies lacked effective external validation. The original studies included in our analysis predominantly utilized a supervised ML process with single-factor + multi-factor LR model selection method and performed internal validation through random sampling (43–45).

In our study, the c-index of LR did not significantly lag behind other types of ML models, which demonstrates relatively high sensitivity and specificity. Hence, we believe that LR exhibits promising predictive potential for PSCI.

In addition, we found that the major modeling variables for the ML-based PSCI prediction models were age, gender, education level, white matter hyperintensity (WMH), stroke history, stroke severity, lesion volume, lesion site, stroke subtype, and vascular risk factors. These modeling variables were still mainly based on past identified risk factors (race, age, gender, education level, vascular risk factor, stroke severity, and stroke lesion site and volume) (46), and very few or no newly identified risk factors were used for modeling, such as blood proteins [homocysteine (Hcy), C-reactive protein (CRP), low-density lipoprotein cholesterol (LDL-C), total cholesterol (TC)] that have been recognized as effective biomarkers for PSCI (47), cognitive reserve (CR) (48), activity and participation of stroke survivors (49), and intestinal dysbiosis (50). Therefore, the newly identified risk factors should be prioritized for further validation as their efficacy as modeling variables remains uncertain.

It was reported that common cognitive screening tools have similar predictive accuracy in PSCI. Although the MoCA has significantly better sensitivity in PSCI prediction than other cognitive screening tools, its specificity is less than desirable (51, 52). This demonstrates that there is a lack of effective prediction models for the early screening of PSCI. However, our findings showed that ML has considerably high predictive accuracy (c-index, sensitivity, and specificity) in PSCI and is a promising tool for predicting PSCI.

A recent systematic review indicated that although PSCI has unique risk factors (e.g., Vascular risk factors, lifestyle, overweight, and obesity), it is currently unclear whether the intervention of these risk factors can effectively reduce the incidence of PSCI. Most approaches for lowering PSCI incidence are still dependent on effective prophylaxis for stroke (46). Therefore, effective prediction tools for the early identification and diagnosis of PSCI are urgently needed. Despite the uncertainty in the intervention measures for post-stroke cognitive functions, some researchers found that physical activity intervention and noninvasive brain stimulation can improve post-stroke cognitive functions compared with conventional care (53). Though, the ≥2-year improvement in PSCI after intervention was small (54). Moreover, patients with cognitive impairment have significantly increased risks of subsequent ischemic and fatal stroke (55, 56). Hence, early identification of appropriate treatment and rehabilitation measures are critical for improving the health and life expectancy of PSCI patients. Our study demonstrated the feasibility of ML in the development of PSCI prediction tools and that ML is also an important means for PSCI prediction.

Given the low number of PSCI prediction models for hemorrhagic stroke included in this study (*n* = 3) (19), the predictive accuracy of ML vs. common cognitive screening tools in PSCI in hemorrhagic stroke patients remains unclear and warrants further investigation.

ML plays an important role in the clinical management of stroke and improvement of the accuracy and efficiency of stroke prediction, diagnosis, personalized treatment, and prognosis assessment (57). For prediction of stroke risk, ML algorithms can be trained using patient data to establish predictive models and estimate the risk of stroke based on individual patient information, clinical indicators, and biomarkers. As for stroke diagnosis, ML can learn and identify radiological features of stroke and assist physicians with early and accurate diagnosis. In addition, ML can predict the efficacy and safety of different treatment options based on the patient’s personal information, medical history, and clinical manifestations, enabling physicians to develop personalized treatment strategies. Furthermore, ML algorithms can predict post-stroke recovery and long-term prognosis based on patients’ clinical and biomarker data. ML has been extensively used in stroke diagnosis, particularly in brain imaging, with SVM being the optimal model for stroke imaging (6, 44, 57). However, in our study, SVM exhibited inferior sensitivity to LR despite higher c-index, and the model size was limited (*n* = 1). Therefore, further exploration and development of SVM in predicting PSCI are warranted. We can attempt to optimize the accuracy of PSCI prediction by using different SVM models and parameter settings. SVM has various variants, such as non-linear SVM, multi-kernel SVM, and support vector regression, which are selected based on specific circumstances. Additionally, the combination of SVM with other ML methods can be explored for PSCI prediction. For instance, integrating SVM with deep learning techniques can improve the accuracy and robustness of predictions when analyzing images or text data. Moreover, extensive clinical validation studies are required to assess the actual effectiveness of SVM in PSCI prediction. The application value of SVM in PSCI prediction can be comprehensively assessed by collecting more data from PSCI patients and evaluating the models on independent validation sets. In conclusion, SVM, as a widely used ML method, has untapped potential in PSCI prediction. Continuous learning and research efforts can further refine and optimize the application of SVM in PSCI prediction, providing more accurate diagnostic and treatment decision support for clinical practitioners.

For this systematic review, the literature search was performed up until December 2022 and additional studies on this topic may become available subsequently. Hence, a regular review of the literature is recommended to obtain the most updated progress on this research topic.

### 4.1. Strengths and limitations

This systematic review is the first to demonstrate the feasibility of ML in PSCI prediction. The included models were highly consistent and were predominantly logistic regression nomograms, which minimized heterogeneity.

Despite a comprehensive literature search, the number of included studies and models was still relatively low, and bias may be present in model construction.

## 5. Conclusion

ML has considerable predictive accuracy and is a promising prediction tool for PSCI. Therefore, future studies should concentrate on constructing ML models based on multi-racial, multi-center, and large-cohort samples and transforming them into simple clinical scoring tools with wide application. This will undoubtedly help with the development of follow-up strategies or rehabilitation measures for stroke patients to reduce their risk of developing cognitive impairment.

## Data availability statement

The original contributions presented in the study are included in the article/Supplementary material, further inquiries can be directed to the corresponding authors.

## Author contributions

All authors listed have made a substantial, direct, and intellectual contribution to the work and approved it for publication.

## Funding

This study was supported by the National Natural Science Foundation of China (31960178 and 82160923), Applied Basic Research Programs of Science and Technology Commission Foundation of Yunnan Province (2019FA007), Key Laboratory of Traditional Chinese Medicine for Prevention and Treatment of Neuropsychiatric Diseases, Yunnan Provincial Department of Education, Scientific Research Projects for High-level Talents of Yunnan University of Chinese Medicine (2019YZG01), Young Top-Notch Talent in 10,000 Talent Program of Yunnan Province (YNWR-QNBJ-2019-235), National Science and Technology Innovation 2030 Major Program (2021ZD0200900), Yunnan Key Research and Development Program (202103AC100005), and Yunnan Province Fabao Gao Expert Workstation Construction Project (202105AF150037).

## Conflict of interest

The authors declare that the research was conducted in the absence of any commercial or financial relationships that could be construed as a potential conflict of interest.

## Publisher’s note

All claims expressed in this article are solely those of the authors and do not necessarily represent those of their affiliated organizations, or those of the publisher, the editors and the reviewers. Any product that may be evaluated in this article, or claim that may be made by its manufacturer, is not guaranteed or endorsed by the publisher.

## Supplementary material

Supplementary Figure S1 Sensitivity analysis of the training set.

Supplementary Figure S2 Sensitivity analysis of the validation set.

Supplementary Figure S3 The funnel plot of the training set.

Supplementary Figure S4 The funnel plot of the validation set.

Supplementary Figure S5 Egger’s regression test of the training set.

Supplementary Figure S6 Egger’s regression test of the validation set.
