# Supplementary material for: Machine learning in the prediction of post-stroke cognitive impairment: a systematic review and meta-analysis
Source: Front Neurol. 2023 Aug 3;14:1211733. doi: 10.3389/fneur.2023.1211733 (PMC10434510; doi:10.3389/fneur.2023.1211733)
Supplement: Supplementary file 1 [file Table_1.DOCX]

**Table S1**: Literature search strategy

**1.Pubmed**

| Search number | Query | Results |
| --- | --- | --- |
| #1 | "Stroke"[Mesh] | 165652 |
| #2 | ((((((((((((((Strokes[Title/Abstract]) OR (Cerebrovascular Accident[Title/Abstract])) OR (Cerebrovascular Accidents[Title/Abstract])) OR (Cerebrovascular Apoplexy[Title/Abstract])) OR (Brain Vascular Accident[Title/Abstract])) OR (Brain Vascular Accidents[Title/Abstract])) OR (Apoplexy[Title/Abstract])) OR (Brain Infarction[Title/Abstract])) OR (Brain Infarctions[Title/Abstract])) OR (Brain Infarct[Title/Abstract])) OR (Brain Infarcts[Title/Abstract])) OR (Brain Venous Infarction[Title/Abstract])) OR (Brain Venous Infarctions[Title/Abstract])) OR (acute cerebrovascular lesion[Title/Abstract])) OR (apoplexia[Title/Abstract]) | 41082 |
| #3 | ("Stroke"[Mesh]) OR (((((((((((((((Strokes[Title/Abstract]) OR (Cerebrovascular Accident[Title/Abstract])) OR (Cerebrovascular Accidents[Title/Abstract])) OR (Cerebrovascular Apoplexy[Title/Abstract])) OR (Brain Vascular Accident[Title/Abstract])) OR (Brain Vascular Accidents[Title/Abstract])) OR (Apoplexy[Title/Abstract])) OR (Brain Infarction[Title/Abstract])) OR (Brain Infarctions[Title/Abstract])) OR (Brain Infarct[Title/Abstract])) OR (Brain Infarcts[Title/Abstract])) OR (Brain Venous Infarction[Title/Abstract])) OR (Brain Venous Infarctions[Title/Abstract])) OR (acute cerebrovascular lesion[Title/Abstract])) OR (apoplexia[Title/Abstract])) | 189513 |
| #4 | "Cognitive Dysfunction"[Mesh] | 32946 |
| #5 | ((((((((((((((((((Cognitive Dysfunctions[Title/Abstract]) OR (Cognitive Impairments[Title/Abstract])) OR (Cognitive Impairment[Title/Abstract])) OR (Mild Neurocognitive Disorder[Title/Abstract])) OR (Mild Neurocognitive Disorders[Title/Abstract])) OR (Cognitive Decline[Title/Abstract])) OR (Cognitive Declines[Title/Abstract])) OR (Mental Deterioration[Title/Abstract])) OR (Mental Deteriorations[Title/Abstract])) OR (cognitive defect[Title/Abstract])) OR (cognition disorder[Title/Abstract])) OR (cognition disorders[Title/Abstract])) OR (cognition disorders[Title/Abstract])) OR (cognitive deficit[Title/Abstract])) OR (cognitive disability[Title/Abstract])) OR (cognitive disorder[Title/Abstract])) OR (cognitive disorders[Title/Abstract])) OR (cognitive dysfunction[Title/Abstract])) OR (cognitive impairment[Title/Abstract]) | 128057 |
| #6 | ("Cognitive Dysfunction"[Mesh]) OR (((((((((((((((((((Cognitive Dysfunctions[Title/Abstract]) OR (Cognitive Impairments[Title/Abstract])) OR (Cognitive Impairment[Title/Abstract])) OR (Mild Neurocognitive Disorder[Title/Abstract])) OR (Mild Neurocognitive Disorders[Title/Abstract])) OR (Cognitive Decline[Title/Abstract])) OR (Cognitive Declines[Title/Abstract])) OR (Mental Deterioration[Title/Abstract])) OR (Mental Deteriorations[Title/Abstract])) OR (cognitive defect[Title/Abstract])) OR (cognition disorder[Title/Abstract])) OR (cognition disorders[Title/Abstract])) OR (cognition disorders[Title/Abstract])) OR (cognitive deficit[Title/Abstract])) OR (cognitive disability[Title/Abstract])) OR (cognitive disorder[Title/Abstract])) OR (cognitive disorders[Title/Abstract])) OR (cognitive dysfunction[Title/Abstract])) OR (cognitive impairment[Title/Abstract])) | 133527 |
| #7 | "Machine Learning"[Mesh] | 51742 |
| #8 | (((((((((((((((((((((((Transfer Learning[Title/Abstract]) OR (Deep learning[Title/Abstract])) OR (Ensemble Learning[Title/Abstract])) OR (artificial intelligence[Title/Abstract])) OR (Prediction model[Title/Abstract])) OR (Risk Prediction[Title/Abstract])) OR (Risk-Prediction[Title/Abstract])) OR (random forest[Title/Abstract])) OR (neural network[Title/Abstract])) OR (neural networks[Title/Abstract])) OR (CNN[Title/Abstract])) OR (Support vector machine[Title/Abstract])) OR (SVM[Title/Abstract])) OR (Gradient Boosting Machine[Title/Abstract])) OR (GBM[Title/Abstract])) OR (Logistic[Title/Abstract])) OR (Nomogram[Title/Abstract])) OR (XGBoost[Title/Abstract])) OR (Decision tree[Title/Abstract])) OR (ResNet-50[Title/Abstract])) OR (ResNet[Title/Abstract])) OR (Naive Bayesian[Title/Abstract])) OR (Radiomics[Title/Abstract])) OR (radiomic[Title/Abstract]) | 631099 |
| #9 | ("Machine Learning"[Mesh]) OR ((((((((((((((((((((((((Transfer Learning[Title/Abstract]) OR (Deep learning[Title/Abstract])) OR (Ensemble Learning[Title/Abstract])) OR (artificial intelligence[Title/Abstract])) OR (Prediction model[Title/Abstract])) OR (Risk Prediction[Title/Abstract])) OR (Risk-Prediction[Title/Abstract])) OR (random forest[Title/Abstract])) OR (neural network[Title/Abstract])) OR (neural networks[Title/Abstract])) OR (CNN[Title/Abstract])) OR (Support vector machine[Title/Abstract])) OR (SVM[Title/Abstract])) OR (Gradient Boosting Machine[Title/Abstract])) OR (GBM[Title/Abstract])) OR (Logistic[Title/Abstract])) OR (Nomogram[Title/Abstract])) OR (XGBoost[Title/Abstract])) OR (Decision tree[Title/Abstract])) OR (ResNet-50[Title/Abstract])) OR (ResNet[Title/Abstract])) OR (Naive Bayesian[Title/Abstract])) OR (Radiomics[Title/Abstract])) OR (radiomic[Title/Abstract])) | 647185 |
| #10 | #3 and #6 and #9 | 386 |

**2.Cochrane**

| Search number | Query | Results |
| --- | --- | --- |
| #1 | MeSH descriptor: [Stroke] explode all trees | 11922 |
| #2 | (‘Strokes’ OR ‘Cerebrovascular Accident’ OR ‘Cerebrovascular Accidents’ OR ‘Cerebrovascular Apoplexy’ OR ‘Brain Vascular Accident’ OR ‘Brain Vascular Accidents’ OR ‘Apoplexy’ OR ‘Brain Infarction’ OR ‘Brain Infarctions’ OR ‘Brain Infarct’ OR ‘Brain Infarcts’ OR ‘Brain Venous Infarction’ OR ‘Brain Venous Infarctions’ OR ‘acute cerebrovascular lesion’ OR ‘apoplexia’ ):ti,ab,kw | 22555 |
| #3 | #1 or #2 | 31253 |
| #4 | MeSH descriptor: [Cognitive Dysfunction] explode all trees | 2394 |
| #5 | (‘Cognitive Dysfunctions’ OR ‘Cognitive Impairments’ OR ‘Cognitive Impairment’ OR ‘Mild Neurocognitive Disorder’ OR ‘Mild Neurocognitive Disorders’ OR ‘Cognitive Decline’ OR ‘Cognitive Declines’ OR ‘Mental Deterioration’ OR ‘Mental Deteriorations’ OR ‘cognitive defect ‘ OR ‘cognition disorder’ OR ‘cognition disorders’ OR ‘cognitive defects’ OR ‘cognitive deficit’ OR ‘cognitive disability’ OR ‘cognitive disorder’ OR ‘cognitive disorders’ OR ‘cognitive dysfunction’ OR ‘cognitive impairment’):ti,ab,kw 53282  #5 MeSH descriptor: [Machine Learning] explode all trees | 53282 |
| #6 | #4 or #5 | 53289 |
| #7 | MeSH descriptor: [Machine Learning] explode all trees | 284 |
| #8 | (‘Transfer Learning’ OR ‘Deep learning’ OR ‘Ensemble Learning’ OR ‘artificial intelligence’ OR ‘Prediction model’ OR ‘Risk Prediction’ OR ‘Risk-Prediction’ OR ‘random forest’ OR ‘neural network’ OR ‘neural networks’ OR ‘CNN’ OR ‘Support vector machine’ OR ‘SVM’ OR ‘Gradient Boosting Machine’ OR ‘GBM’ OR ‘Logistic’ OR ‘Nomogram’ OR ‘XGBoost’ OR ‘Decision tree’ OR ‘ResNet-50’ OR ‘ResNet’ OR ‘Naive Bayesian’ OR ‘Radiomics’ OR ‘radiomic’):ti,ab,kw | 44375 |
| #9 | #7 or #8 | 44438 |
| #10 | #3 and #6 and #9 | 95 |

**3.Embase**

| Search number | Query | Results |
| --- | --- | --- |
| #1 | 'cerebrovascular accident'/exp | 402881 |
| #2 | 'strokes':ti,ab,kw OR 'stroke':ti,ab,kw OR 'cerebrovascular accidents':ti,ab,kw OR 'cerebrovascular apoplexy':ti,ab,kw OR 'brain vascular accident':ti,ab,kw OR 'brain vascular accidents':ti,ab,kw OR 'apoplexy':ti,ab,kw OR 'brain infarction':ti,ab,kw OR 'brain infarctions':ti,ab,kw OR 'brain infarct':ti,ab,kw OR 'brain infarcts':ti,ab,kw OR 'brain venous infarction':ti,ab,kw OR 'brain venous infarctions':ti,ab,kw OR 'acute cerebrovascular lesion':ti,ab,kw OR 'apoplexia':ti,ab,kw | 498791 |
| #3 | #1 OR #2 | 602402 |
| #4 | 'cognitive defect'/exp | 585225 |
| #5 | 'cognitive dysfunctions':ti,ab,kw OR 'cognitive impairments':ti,ab,kw OR 'mild neurocognitive disorder':ti,ab,kw OR 'mild neurocognitive disorders':ti,ab,kw OR 'cognitive decline':ti,ab,kw OR 'cognitive declines':ti,ab,kw OR 'mental deterioration':ti,ab,kw OR 'mental deteriorations':ti,ab,kw OR 'cognition disorder':ti,ab,kw OR 'cognition disorders':ti,ab,kw OR 'cognitive defects':ti,ab,kw OR 'cognitive deficit':ti,ab,kw OR 'cognitive disability':ti,ab,kw OR 'cognitive disorder':ti,ab,kw OR 'cognitive disorders':ti,ab,kw OR 'cognitive dysfunction':ti,ab,kw OR 'cognitive impairment':ti,ab,kw | 194837 |
| #6 | #4 OR #5 | 621484 |
| #7 | 'machine learning'/exp | 350581 |
| #8 | 'transfer learning':ti,ab,kw OR 'deep learning':ti,ab,kw OR 'ensemble learning':ti,ab,kw OR 'artificial intelligence':ti,ab,kw OR 'prediction model':ti,ab,kw OR 'risk prediction':ti,ab,kw OR 'risk-prediction':ti,ab,kw OR 'random forest':ti,ab,kw OR 'neural network':ti,ab,kw OR 'neural networks':ti,ab,kw OR 'cnn':ti,ab,kw OR 'support vector machine':ti,ab,kw OR 'svm':ti,ab,kw OR 'gradient boosting machine':ti,ab,kw OR 'gbm':ti,ab,kw OR 'logistic':ti,ab,kw OR 'nomogram':ti,ab,kw OR 'xgboost':ti,ab,kw OR 'decision tree':ti,ab,kw OR 'resnet-50':ti,ab,kw OR 'resnet':ti,ab,kw OR 'naive bayesian':ti,ab,kw OR 'radiomics':ti,ab,kw OR 'radiomic':ti,ab,kw | 878378 |
| #9 | #7 OR #8 | 1089957 |
| #10 | #7 AND #8 AND #9 | 2775 |

**4.Web of science**

| Search number | Query | Results |
| --- | --- | --- |
| #1 | Stroke (Topic) OR Strokes (Topic) OR Cerebrovascular Accident (Topic) OR Cerebrovascular Accidents (Topic) OR Cerebrovascular Apoplexy (Topic) OR Brain Vascular Accident (Topic) OR Brain Vascular Accidents (Topic) OR Apoplexy (Topic) OR Brain Infarction (Topic) OR Brain Infarctions (Topic) OR Brain Infarct (Topic) OR Brain Infarcts (Topic) OR Brain Venous Infarction (Topic) OR Brain Venous Infarctions (Topic) OR acute cerebrovascular lesion (Topic) OR apoplexia (Topic) | 448848 |
| #2 | Cognitive Dysfunction (Topic) OR Cognitive Dysfunctions (Topic) OR Cognitive Impairments (Topic) OR Cognitive Impairment (Topic) OR Mild Neurocognitive Disorder (Topic) OR Mild Neurocognitive Disorders (Topic) OR Cognitive Decline (Topic) OR Cognitive Declines (Topic) OR Mental Deterioration (Topic) OR Mental Deteriorations (Topic) OR cognitive defect (Topic) OR cognition disorder (Topic) OR cognition disorders (Topic) OR cognitive defects (Topic) OR cognitive deficit (Topic) OR cognitive disability (Topic) OR cognitive disorder (Topic) OR cognitive disorders (Topic) OR cognitive dysfunction (Topic) OR cognitive impairment (Topic) | 372199 |
| #3 | machine learning (Topic) OR Transfer Learning (Topic) OR Deep learning (Topic) OR Ensemble Learning (Topic) OR artificial intelligence (Topic) OR Prediction model (Topic) OR Risk Prediction (Topic) OR Risk-Prediction (Topic) OR random forest (Topic) OR neural network (Topic) OR neural networks (Topic) OR CNN (Topic) OR Support vector machine (Topic) OR SVM (Topic) OR Gradient Boosting Machine (Topic) OR GBM (Topic) OR Logistic (Topic) OR Nomogram (Topic) OR XGBoost (Topic) OR Decision tree (Topic) OR ResNet-50 (Topic) OR ResNet (Topic) OR Naive Bayesian (Topic) OR Radiomics (Topic) OR radiomic (Topic) | 2491183 |
| #4 | #1 and #2 and #3 | 1797 |
