# Supplementary figures and images for: Machine learning in the prediction of post-stroke cognitive impairment: a systematic review and meta-analysis
Source: Front Neurol. 2023 Aug 3;14:1211733. doi: 10.3389/fneur.2023.1211733 (PMC10434510; doi:10.3389/fneur.2023.1211733)

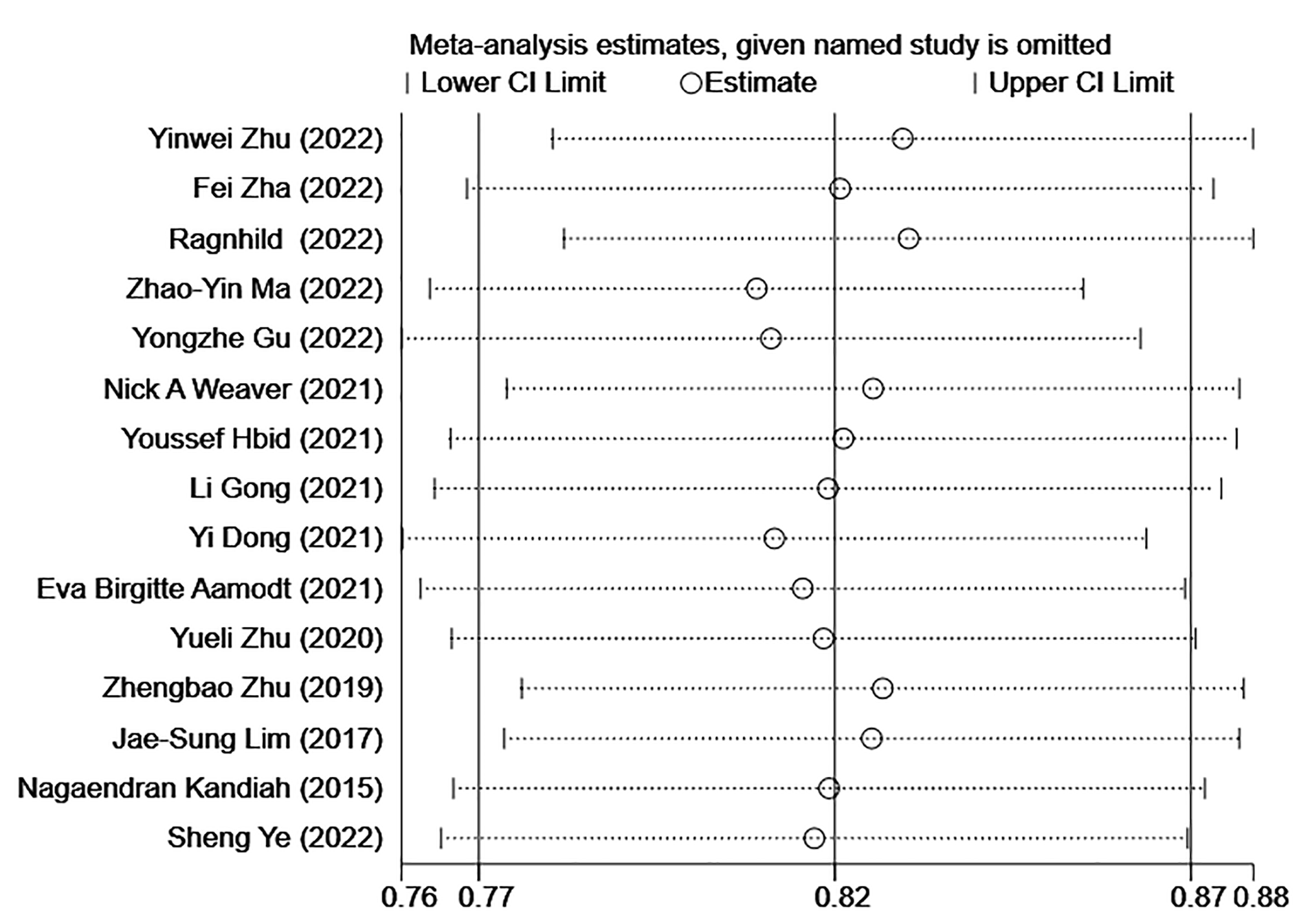

Supplement: Supplementary Figure S1 — Sensitivity analysis of the training set. [file Image_1.TIF]

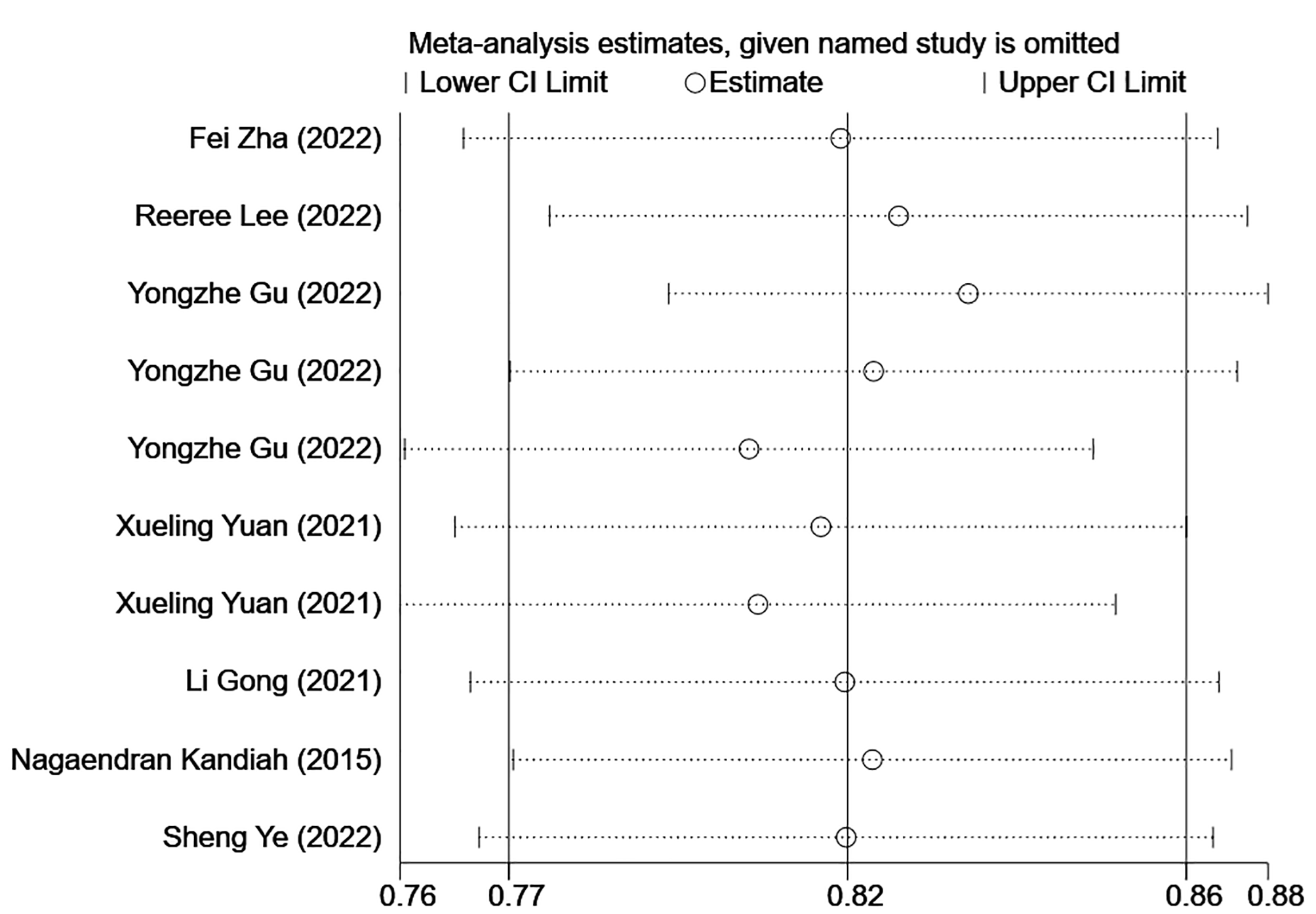

Supplement: Supplementary Figure S2 — Sensitivity analysis of the validation set. [file Image_2.TIF]

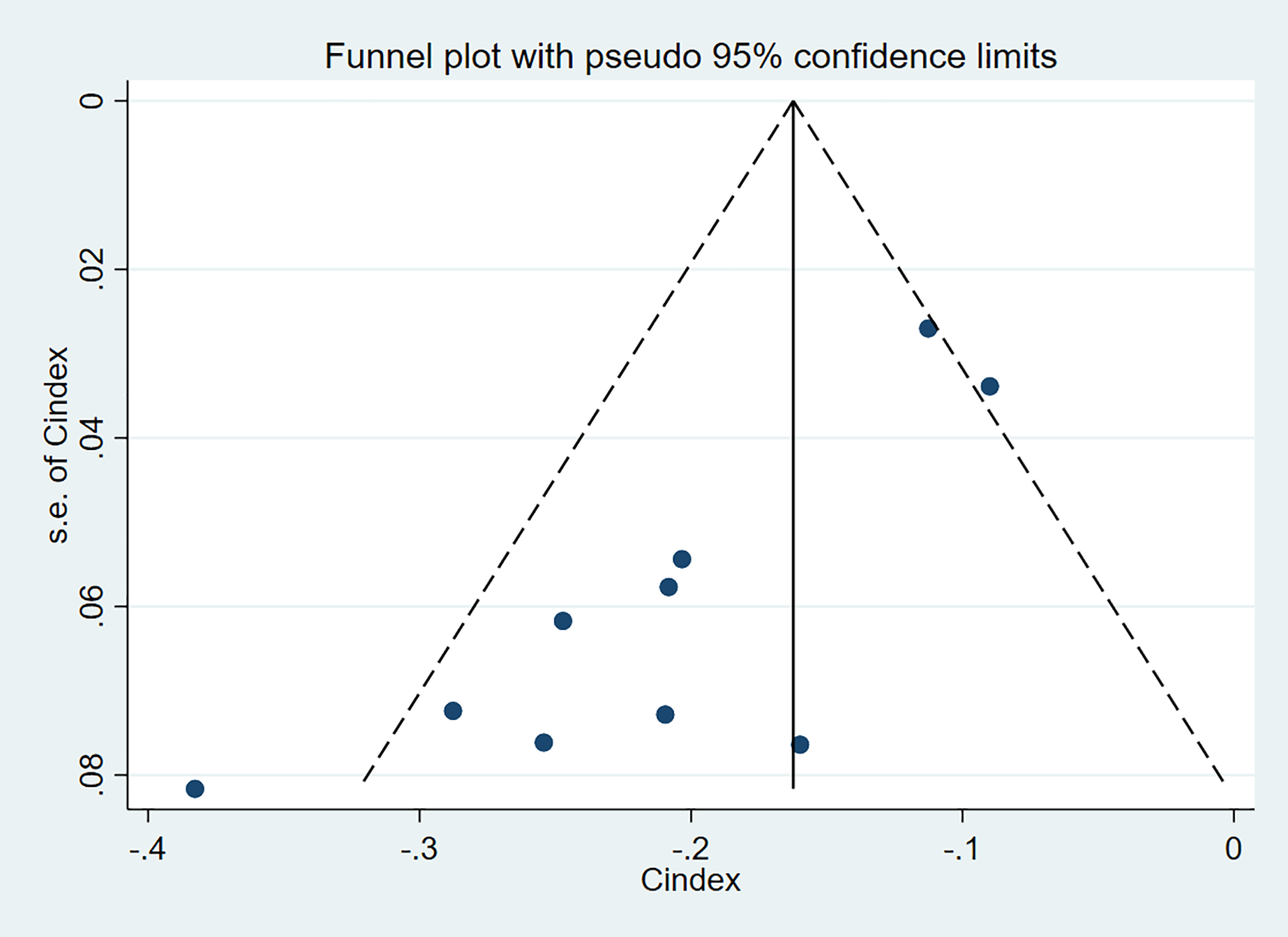

Supplement: Supplementary Figure S3 — The funnel plot of the training set. [file Image_3.TIF]

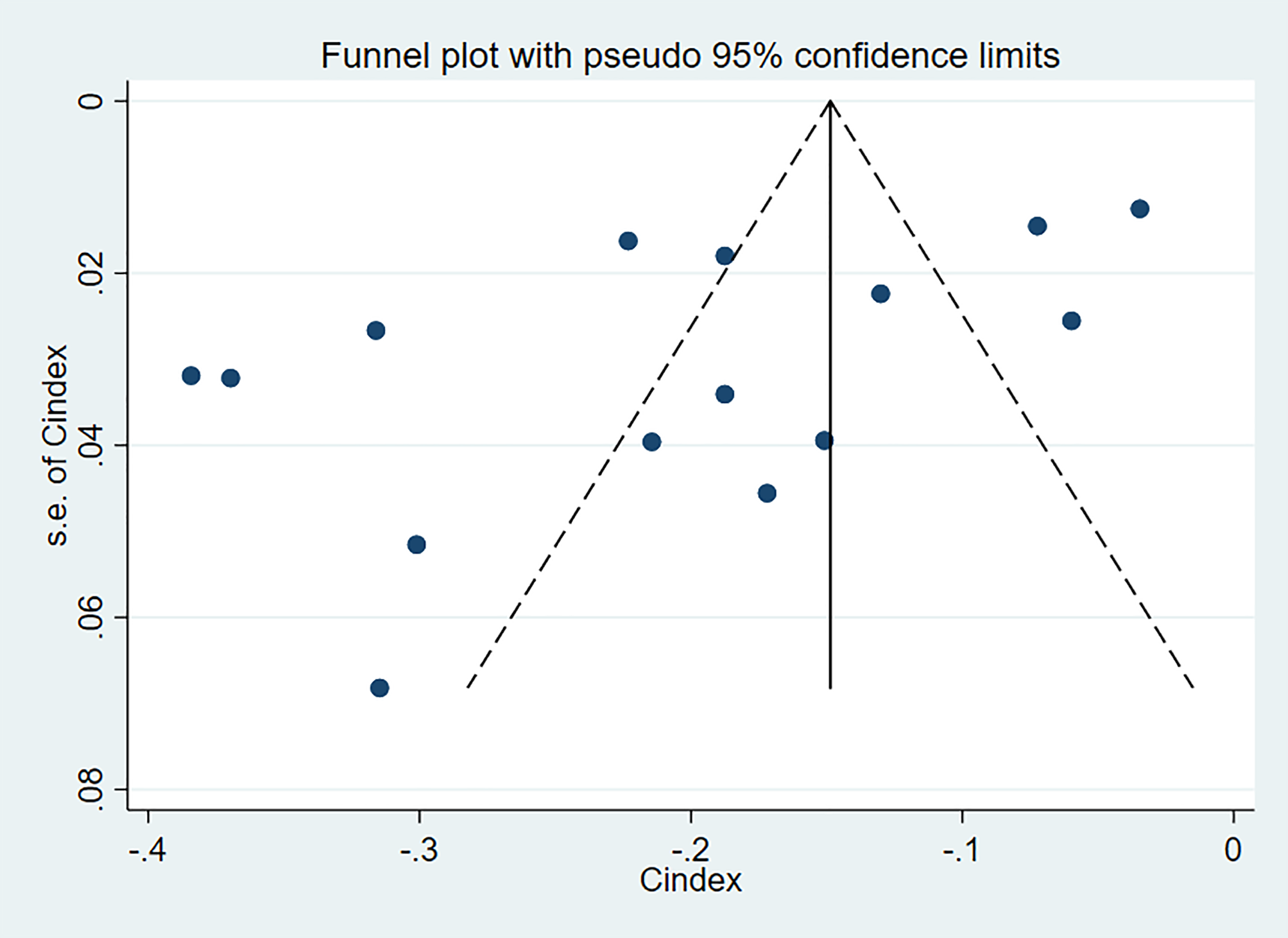

Supplement: Supplementary Figure S4 — The funnel plot of the validation set. [file Image_4.TIF]

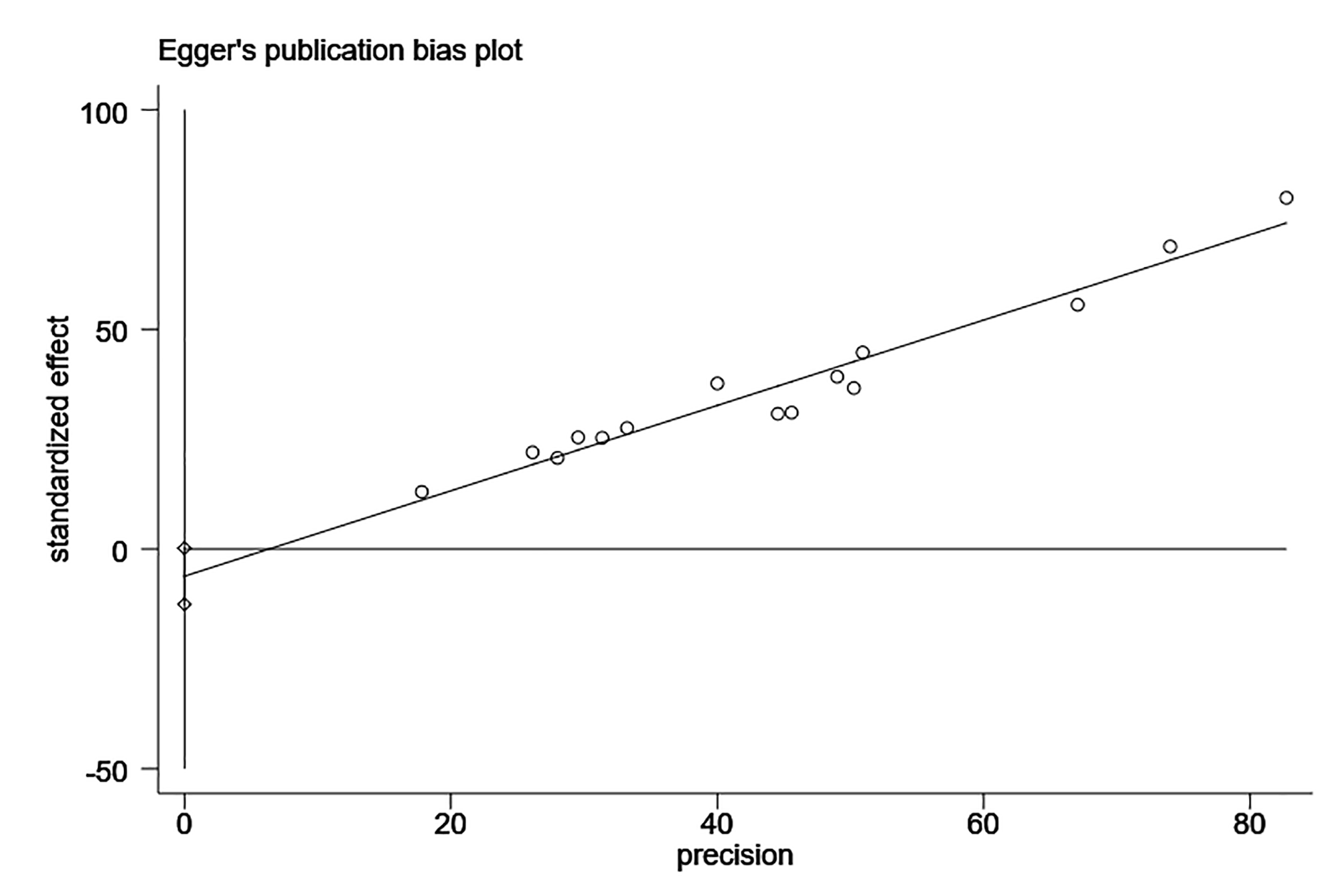

Supplement: Supplementary Figure S5 — Egger’s regression test of the training set. [file Image_5.TIF]

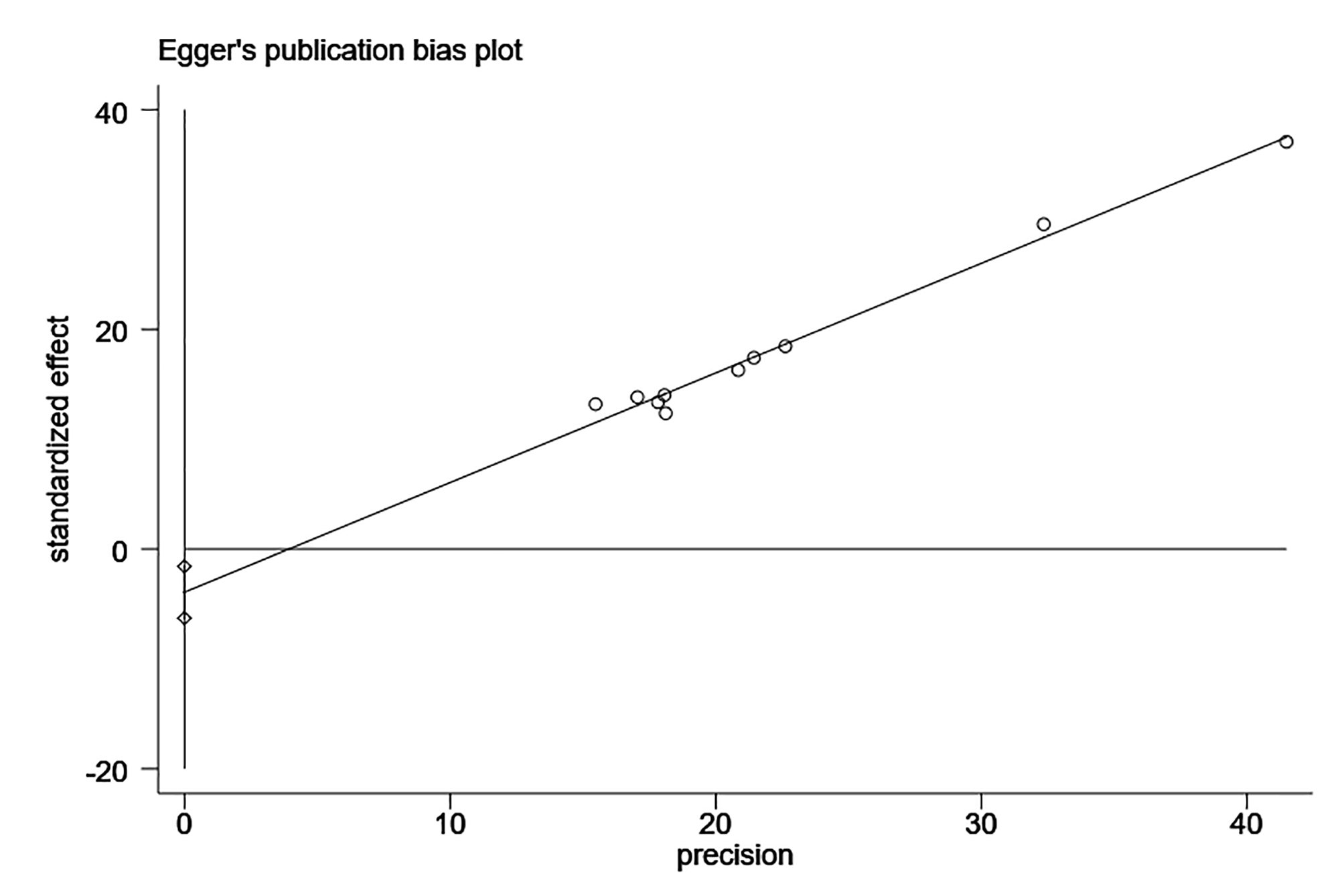

Supplement: Supplementary Figure S6 — Egger’s regression test of the validation set. [file Image_6.TIF]
